# The Effectiveness of Interventions Targeting Adolescents in HPV Vaccination—A Scoping Review

**DOI:** 10.3390/medicina60091550

**Published:** 2024-09-22

**Authors:** Camelia Florina Iova, Lucia Georgeta Daina, Mădălina Diana Daina, Timea Claudia Ghitea

**Affiliations:** 1 Faculty of Medicine and Pharmacy, Doctoral School, University of Oradea, 410081 Oradea, Romania; camyowa@yahoo.com (C.F.I.); diana_daina98@yahoo.com (M.D.D.); 2 Department of Psycho-Neurosciences and Recovery, Faculty of Medicine and Pharmacy, University of Oradea, 410081 Oradea, Romania; lucidaina@gmail.com; 3 Pharmacy Department, Faculty of Medicine and Pharmacy, University of Oradea, 410081 Oradea, Romania

**Keywords:** adolescents, educational interventions, peer education, multicomponent interventions

## Abstract

Adolescents are the target group for HPV vaccination. Studies that examine factors influencing acceptability among adolescents and interventions aimed at improving knowledge, attitudes, perceptions, intentions, and, most importantly, vaccination rates are less common than those addressing parents or healthcare professionals. The specialized literature was searched for studies evaluating the impact of various interventions on adolescents. In the final analysis, 41 studies were included (35 original studies and 6 reviews). Educational interventions increased adolescents’ knowledge scores in the selected studies. Peer education proved highly effective in rapidly and significantly improving knowledge about HPV. Additionally, multicomponent interventions generated awareness and knowledge that persisted for months after the interventions. HPV vaccine uptake increased following educational interventions in 11 out of the 14 studies that evaluated this outcome; studies presenting multicomponent interventions also proved effective in improving vaccination rates. Higher HPV vaccine series completion was reported following a reminder system strategy. Interventions directed at adolescents, combined with strategies involving parents and healthcare professionals, can play an important role in improving HPV vaccination rates. Educated adolescents must be involved in decisions about their own health and can be a valuable source of information for their peers and parents.

## 1. Introduction

Vaccination against human papillomavirus (HPV) is crucial in significantly reducing the risk of HPV infections, thereby preventing not only cervical cancer, but also other types of cancer [1].

The literature abounds in studies focused on identifying the factors that influence the acceptability of HPV vaccination among parents, but there are few articles that deal with this topic from the perspective of the target group: adolescents. On the other hand, the importance of involving adolescents in decisions regarding health in general and in those regarding vaccination in particular is increasingly highlighted [1].

The adolescent stage is challenging because adolescents often do not respond well to authority or attempts to impose rules, including medical ones. At the same time, young people at this stage are more likely to engage in behaviors that could increase their risk of developing various forms of cancer later in life [2,3].

Adolescents are often left out of vaccination decisions due to their age, limited understanding of vaccination, and challenges in communication among parents, adolescents, and healthcare professionals [4,5]. Also, very little information is directly transmitted to adolescents. Studies emphasize the need to educate and inform the target group, as well as the fact that adolescents want to obtain information and be part of the decision-making process [6].

If parents’ lack of knowledge about vaccines can hinder the immunization of adolescents, studies show an even lower level of knowledge among adolescents themselves [7]. Adolescents do not receive information/are not educated about HPV infection or HPV vaccination on a regular basis, so they generally have little knowledge, especially when it comes to associating the infection with cervical cancer [8]. For this reason and in this context, adolescence could be an ideal time for educational interventions, both at school and within the community. Educated adolescents can be a valuable source of information for parents, especially in communities with low socioeconomic levels [3].

Identifying interventions that increase the acceptability of HPV vaccination and, by extension, vaccination coverage, is a priority for all public health organizations involved in prevention [9]. Besides interventions targeted at parents and healthcare professionals, strategies involving the target group could be crucial in boosting vaccine acceptability. Educational interventions can enhance the acceptability of HPV vaccination by increasing awareness of the risks and severity of infection and associated diseases (such as cervical cancer) and by dispelling prejudices and myths related to vaccination [10].

Few articles in the literature propose interventions that directly address the target population for HPV vaccination. Most of them propose strategies that address parents.

The purpose of this review was to evaluate the specialized literature and assess the impact of various interventions addressed primarily and directly to adolescents, on HPV vaccination uptake and intentions, but also on knowledge, perceptions, and attitudes related to the HPV vaccine, HPV infections, and its associated cancers.

## 2. Materials and Methods

### 2.1. Outcomes

This review aimed to determine whether there was an increase in knowledge levels, positive changes in perceptions, attitudes, and intentions related to vaccination, as well as vaccine acceptability, and implicitly the rates of vaccination initiation or completion after different types of interventions that targeted adolescents. Thus, the primary outcome was to follow up on vaccination rates in adolescents included in the studies (initiation or full vaccination), and the secondary outcomes were to follow up on whether or not the level of knowledge, attitudes and intentions related to vaccination changed.

### 2.2. Inclusion and Exclusion Criteria

Inclusion criteria: studies that included only adolescents or “also adolescents” (according to the WHO definition, individuals aged 10–19 years), regardless of the country of implementation, articles that also have an English version available, articles that described and presented the results of different types of interventions (educational, reminders, incentives, multicomponent), original articles and well-structured systematic reviews in terms of results. All study designs were accepted if they met the inclusion criteria.

Exclusion criteria: abstracts, irrelevant articles, articles that included only medical professionals or parents, articles without intervention or with intervention, but without presenting its effects (study protocols), articles unrelated to the subject, articles that could not be obtained in full-text versions, editorials, comments, articles presenting legislative interventions such as self-consent or mandatory vaccination.

### 2.3. Search Strategy

The search followed the Population, Intervention, Comparison, Outcome (PICO) model. In this model, P represents the population (adolescents), I represent the intervention or strategy, C represents the comparison group (if applicable), and O represents the outcomes such as acceptability of the intervention, knowledge, attitude change, intentions, and HPV vaccination rates. Keywords and combinations of them were utilized, based on 6 criteria: “HPV vaccination”, “adolescents/teenagers/teens”, and “interventions/strategies”, separated by the operator “OR”. PubMed, Web of Science, and Scopus databases were searched for relevant articles. A Google and Google Scholar search was included, to avoid missing studies relevant to the topic addressed.

### 2.4. Study Selection

First, all the titles were read, then the abstracts, and finally the full-text articles. After reviewing the articles and removing duplicates, a list of articles to be included in this study was established based on pre-established criteria. The required data, first author, year of publication, strategy described, country of implementation, target population, study design, sample size, objectives, and results, were collected and entered into an Excel file.

### 2.5. Mixed-Methods Appraisal Tool (MMAT)

The Mixed-Methods Appraisal Tool (MMAT) is a widely used tool for assessing the methodological quality of different types of studies, including qualitative, quantitative, and mixed-methods research. It allows for the evaluation of each study type based on five key criteria, providing a structured approach to determining the reliability and rigor of studies in a systematic or scoping review. The MMAT does not recommend assigning a total score but instead focuses on the number of criteria met, which gives a clear indication of the study’s overall quality.

Table 1 summarizes the quality assessment of studies included in the review, classified by study type, the number of criteria met, and overall quality judgment. Among qualitative studies, 5 (12.20%) were rated as moderate quality, meeting 3 out of 4 criteria, while 8 (19.51%) were deemed high quality, meeting 4 out of 5 criteria. In the category of quantitative randomized controlled trials (RCTs), 6 studies (14.63%) were of moderate quality, and 3 studies (7.32%) were assessed as high quality, meeting 3 and 4 out of 5 criteria, respectively. Mixed-methods studies comprised a significant portion, with 6 (14.63%) of moderate quality and 13 (31.71%) of high quality, based on 3 and 4 out of 5 criteria. In total, 41 studies were assessed, providing a comprehensive overview of the methodological quality across different study types, the review contains a significant proportion of high-quality studies, particularly in the mixed-methods category, reflecting a strong representation of this research design.

### 2.6. Statistical Analysis

This study had an exit rate of 0.00%. Chi-square tests were used to identify statistically significant lots (with Asymp. Sig. < 0.05). Statistical analysis was conducted using SPSS Statistics version 20.0 (Armonk, NY, USA), and included the calculation of mean values, frequency intervals, standard deviations, and statistical significance using the Student’s *t*-test. The distribution of the lots was found to approximate a normal distribution based on numerical data assumptions. The Bravais–Pearson correlation coefficient was used to assess the relationship between two variables independently of their measurement units. A significance level of *p* < 0.05 was set for ANOVA, with *p* < 0.01 indicating a highly significant result. Bonferroni post hoc analysis was applied for subgroup comparisons between groups.

## 3. Results

Following the search of the PubMed, Web of Science, and Scopus databases, a total of 5166 articles were identified (3745 in PubMed, 536 in Web of Science, and 885 in Scopus). After removing duplicates, 3353 articles were analyzed (analysis of abstracts or full-text article where needed) and 3124 were excluded; the remaining 229 articles were analyzed in terms of inclusion and exclusion criteria; articles that have been proven, following the full-text review, to evaluate the effects of vaccination programs at the population level, analyze the cost/efficiency ratio of vaccination, present interventions addressed/focused on parents, articles without interventions, assessing the level of knowledge and attitudes related to HPV vaccination or articles presenting models for increasing the acceptability of vaccination or study protocols, were excluded.

**Figure 1:**
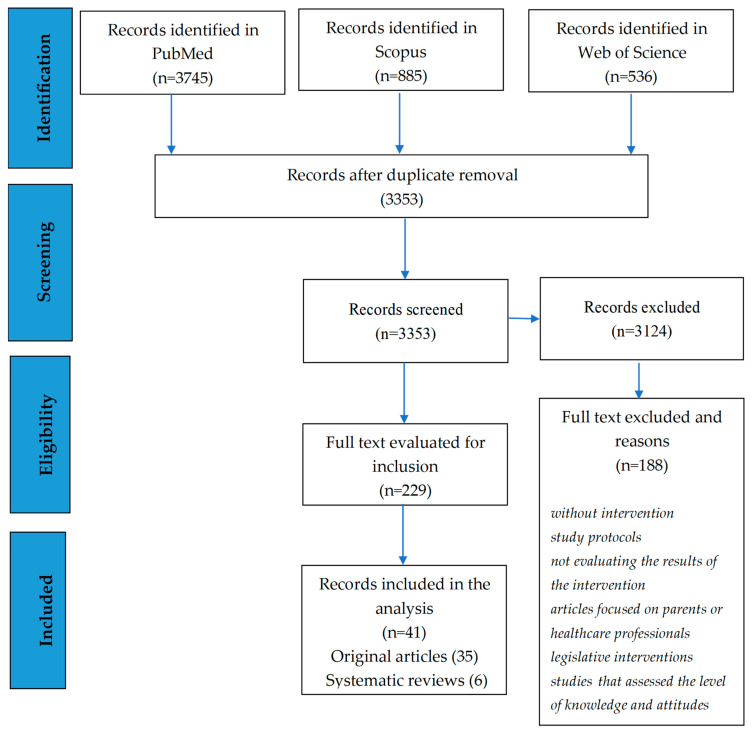
Flow chart of article selection process [11].

In the final analysis, 41 articles were included 35 original articles that presented interventions that targeted adolescents and 6 systematic reviews that displayed different studies on strategies meant to improve vaccination uptake that tackled, aside from parents and healthcare professionals, also adolescents (Table 2 and Figure 1).

### 3.1. General Characteristics of the Original Studies

Out of the 35 original studies, most were conducted in 2015 (5), 2018 (5), 2019 (5), 2022 (4) and 2020 (3), respectively. Therefore, most of the original studies included in this analysis (20 out of 35–57.14%) were published starting with 2018 (2018–2023). The majority of the original studies, were conducted in Europe—13 studies (4 in Italy, 2 in the UK, 2 in Sweden, and one each in the Netherlands, Germany, Greece, Hungary, and Norway) and North America—12 studies (USA), with 5 in Asia, 1 in Australia, 1 in South Africa, 1 in South America (Brazil), and 2 in West Africa (Nigeria). Information on the design of the study was not always provided.

The locations of the studies were different, but in most cases (25 out of 35 studies—71.43%), the interventions were conducted and implemented in schools (middle schools, secondary schools, high schools, vocational and technical schools, elementary schools), the others in various other locations (medical offices or clinics, local fairs, restaurants or even in the investigator’s car) or at home, via the internet.

The target population for the interventions, as intended, was solely adolescents (girls or girls and boys) in 22 (62.86%) of the original articles were analyzed. In another seven studies, parents were also approached, and in four studies, both adult women and young adults were included. Additionally, two studies targeted aside from adolescents, parents, and healthcare providers. From all the studies, we collected data to assess the impact of these interventions on adolescents.

### 3.2. General Characteristics of the Systematic Reviews

The systematic reviews (6) were published between 2014 and 2020. Out of the systematic reviews examined, most studies (51) were included in the one conducted by Walling et al. [12]. Except for the review conducted by Flood et al., which only included studies focused on adolescents, the others followed interventions and their effects on adolescents, parents, and healthcare professionals [13]. Acampora et al. analyzed 38 studies; 21 evaluated interventions targeting parents and adolescents; among these, 5 were reminder-based strategies, 7 involved education/information and communication strategies and 9 were multicomponent interventions [11]. Abdullahi et al. included 16 studies in the systematic review, of which 7 targeted only adolescents, 5 mixed studies, and the others targeted parents, and medical professionals [14]. Walling et al. analyzed 51 studies, including 2 informational interventions, 18 behavioral interventions, and 31 environmental interventions [12]. In addition, Fu et al. reviewed 33 studies on educational interventions, with 8 of them specifically evaluating outcomes in adolescents and young adults [15]. Francis et al. conducted a systematic review that included studies evaluating the effects of interventions using different communication technologies (text messages, automated phone calls, emails, interactive videos) on HPV vaccination rates. Out of the 12 studies, 5 were focused on patients [16]. Flood et al. conducted a systematic review of studies evaluating different school-based educational interventions in adolescents aged 15–17 years. They tracked post-intervention HPV vaccination rates, knowledge, and attitudes related to HPV and associated cancers [13].

### 3.3. Types of Interventions in the Original Studies

Out of 35 interventions, 30 were educational, of which 3 were peer-education studies, 4 were multicomponent–educational plus reminder (2), educational plus reminder plus incentives (1), educational and logistical (1), and 1 intervention was based only on reminder strategy.

#### 3.3.1. Educational Strategies/Interventions

Some studies presented one educational intervention, others proposed complex educational strategies, implemented in different forms: face-to-face sessions, interviews (Lai et al., Grandahl et al.) [8,17], courses, lectures (Esposito et al., Ifediora et al.) [7,18], lessons and seminars (Costantino et al., Gotvall et al., Marek et al.) [19,20,21], power point presentations (Zhang et al., Kwan et al., Costantino et al., Mbulawa et al., Liu et al.) [19,22,23,24,25], held by health education teachers (Costantino et al., Marek et al.) [19,21], doctors and researchers (Zhang et al.) [22], nurses (Grandahl et al., Gotvall et al.) [8,20], trained university student (Gotvall et al.) [20], some followed by question and answer sessions, videogames (Cates et al.) [26], mobile games (Fight HPV—Ruiz Lopez et al.) [27], comic books (Shin et al.) [28], role-play technique presentation (the VacciniAmo le scuole project—Poscia et al.) [29], specifically made books (Ifediora et al.) [18], films/audiovisual presentations (Tu et al., Brabin et al., Thanasas et al.) [30,31,32], video presentations with testimonials and adolescents receiving the vaccines (Sanderson et al.) [33], storytelling narrative videos (Lee et al.) [34], leaflets with information about HPV (Wegwarth et al., Grandahl et al., Hofman et al., Lloyd et al.) [8,35,36,37], booklets (Suryadevara et al.) [38], flipcharts with pictures, printed message cards—“Piss Off, HPV!” (Ferreira et al.) [39], banners (Mbulawa et al.) [24], web-based educational programs (Esposito et al., Gotvall et al., Starling et al.—GoHealthy Girls) [7,20,40], social media interventions—Facebook-assisted teaching methods (Lai et al.) [17], Facebook page (About Your Health—Ortiz et al.) [41], and workshops (Ifediora et al.) [18].

Cates et al. introduced a game called “Land of Secret Gardens” centered around the HPV vaccine. In the game, the vaccine is portrayed as a magic potion, and teenagers encounter messages about HPV and vaccination as they progress through the game [26].

Shin et al. proposed a comic book that tells the story of a teenage girl who learns about the HPV vaccine at school. She then discusses HPV vaccines with her peers and family. The purpose of the comic book was to evaluate its impact on HPV and HPV vaccine knowledge, beliefs, and intentions [28,42].

Esposito et al. conducted a study to assess the impact of web-based educational interventions providing information on vaccine-preventable diseases and vaccination on vaccination coverage in adolescents aged 11–18 years. The study involved three groups: a control group, a group with access to a website dedicated to vaccines and vaccination, and a third group that, in addition to the website, participated in a course led by experts in the field [7].

Similarly, Gottvall et al. proposed a comprehensive educational intervention in the form of a course on HPV and prevention methods, with a focus on HPV vaccination and condom use. The study also directed teens to a website with information about HPV and other STIs. On this website, students were given the opportunity to answer questions about HPV, with the first 10 correct respondents being rewarded with a cinema ticket [20].

Starling et al. developed an interactive website called GoHealthyGirls, organized into four sections; the third module, Info for Teens, was created specifically for teenagers and was designed as an interactive game with questions about HPV and HPV vaccination, a text message simulator and a frequently asked questions section [40].

Ruiz-Lopez et al. developed FightHPV, a mobile game aimed at raising awareness about the importance of HPV vaccination in preventing cervical cancer. The game provides information about how the virus can persist and transform normal tissue into precancerous lesions and cancer. It also highlights the risk factors, such as unprotected sexual intercourse, and educates players about cervical cancer vaccination and screening [27].

Ferreira et al. implemented an educational initiative called “Piss off, HPV!” involving the distribution of printed message cards about HPV vaccination [39].

Lai et al. developed an educational program on cervical cancer prevention conducted through both a social network (Facebook) and face-to-face discussions [17]. Ortiz et al. also created a private Facebook page called “About Your Health” and posted health information, including details about HPV and HPV vaccination [41].

Other studies proposed intervention in the form of PowerPoint presentations (Zhang et al., Kwan et al., Mbulawa et al., Liu et al.) [22,23,24,25]. Costantino et al. also conducted a multicenter project that comprised an educational intervention—a 20 min lesson and slide presentation [19].

Gain-framed messages, highlighting the benefits of vaccination, and loss-framed messages, showing the risks/costs of non-vaccination delivered to adolescents by Tu et al. in the form of a 12 and 13 min film to follow the change in the level of knowledge and intention to vaccinate against HPV, respectively [30]. Similarly, in the study conducted by Sanderson et al., adolescents and their parents watched a video featuring images of adolescents receiving the HPV vaccine, as well as testimonials from parents and medical professionals; they also received educational materials in the form of brochures/flyers [33].

Brabin et al. also utilized an educational film to communicate information about HPV infection, vaccination, and cervical cancer to a group of adolescent girls and to evaluate its impact on them [31]. Lee et al. developed an educational program dedicated to mothers and their teenage daughters (mother and daughter dyads), in the form of storytelling narrative videos entitled “Save My Daughter from Cervical Cancer: Stories by Khmer Mothers and Daughters”. The intervention included information on how the community managed to overcome barriers related to HPV vaccination and testimonials from already vaccinated girls, who presented the factors that influenced their decision [34].

Thanasas et al. assessed the impact of an educational intervention in changing adolescents’ knowledge and acceptability of HPV vaccination; the adolescents in the intervention group participated in a lecture that covered information about the risks and benefits of vaccination, how to access vaccination services, eligible ages, but also about HPV infection [32].

The intervention led by Marek et al. took place during class hours and included information about HPV infection, the female reproductive system, risk factors for cervical cancer, and prevention methods. The presentation was followed by a Q&A session and the students received handouts with key messages on the topic [21].

Ifediora et al. initiated a campaign targeting teenagers, which was carried out under the slogan “Arm our Youths (ArOY) Health Campaign”. Workshops were conducted to train all the participating teachers, ensuring a standardized approach. The intervention consisted of courses on cervical cancer, covering risk factors, symptoms, vaccination, and screening. A dedicated curriculum was developed and questions from the intervention courses were included in the tests and exams for the Civic Education discipline, contributing to the final grade. Specially designed books were donated to all the schools participating in the study to serve as an additional source of information [18].

Poscia et al. set up a project—VacciniAmo le Scuole—in schools across three Italian regions, a project in which local public health authorities were also involved. The project included organizing meetings with students from each class, where promotional and educational activities were carried out, featuring both theoretical and interactive components, such as role-play techniques [29].

The educational intervention proposed by Grandahl et al. was structured according to the Health Belief Model and consisted of a face-to-face presentation, a flipchart, and a leaflet in which they addressed the mode of transmission, diseases caused by HPV infection, risk factors, prevention, locations for vaccination, and the importance of cervical cancer screening [8].

Adolescents received leaflets and booklets about HPV, HPV vaccination, and cervical cancer in the studies conducted by Wegwarth et al., Hofman et al., Lloyd et al., and Suryadevara et al. [35,36,37,38]. Wegwarth et al. analyzed the effects of an educational intervention carried out according to the principle of balanced risk communication. Thus, they assigned two groups: a group that received a pamphlet/booklet with balanced information about vaccination-balanced leaflet group and the group that received a pamphlet/booklet designed by an anti-cancer organization, which did not meet the balanced risk communication criteria-unbalanced leaflet group [35].

#### 3.3.2. Peer Education Interventions

Involving other adolescents/young people in the education of those of similar ages—peer education, can be an effective strategy because adolescents feel more comfortable discussing certain issues with those of their kind, provided that young people are properly trained [13].

A total of three original studies (Sadoh et al., Ferrara et al., Rane et al.) proposed peer health education strategies. In the study conducted by Sadoh et al., the authors trained adolescents, who in turn sent messages about HPV and cervical cancer to their peers using flyers/brochures. Ferrara et al. also selected and trained students during a series of meetings and workshops where they learned about HPV infection, HPV vaccination, HPV testing and cervical cancer screening, risk behaviors, but also about teamwork, and ways of communicating information. In the study led by Rane et al., adolescent champions were trained and provided information about HPV vaccination and the impact of HPV infection on health; the educational messages were adapted to the school environment [2,43,44].

#### 3.3.3. Multicomponent Interventions/Strategies

Mohanty et al. proposed reaching out to adolescents through social media with an information campaign called 3 for ME on Facebook. The campaign aimed to address adolescents’ questions and misinformation about vaccines. To make the vaccine more accessible, it was offered free of charge, and teenagers were also sent reminder letters as part of the campaign. The campaign allowed teenagers to find vaccination locations at medical centers or community events such as health fairs and basketball tournaments [45].

Davies et al. (Skinner et al., Davis et al., 2021, Cooper et al.) developed HPV.edu, a complex school-based intervention with three major components: (1) adolescent intervention—interactive lesson and watching an animated film about HPV and HPV vaccination, a take-home magazine, a website (http://takechargehpv.org, accessed on 26 August 2024) and a mobile app; distraction/relaxation methods such as iPads in the waiting rooms were also used (2) a HPV vaccination decision support tool for adolescents and parents and (3) logistical strategies, designed to increase consent form return rates (direct mails addressed to parents, reminders, incentives) [4,5,6,46].

The Girls OnGuard project, designed by DiClemente et al., aimed to assess vaccination compliance in adolescents; participants in the intervention group were exposed to a 12 min interactive computer-delivered media presentation on HPV vaccination and also received a motivational keychain to store a vaccine reminder card [47].

In the study conducted by Mantzari et al., all participating adolescents were sent invitations (including date and location) to receive the first dose of the HPV vaccine. Along with the invitations, they were given a leaflet containing information about HPV and vaccination Those who desired more information were directed to the website of the National Public Health Authority. Adolescents in the intervention group were offered shopping vouchers worth 52 euros for completing all 3 doses of the HPV vaccine (23 euros for the first dose, 6 euros for the second dose, and another 23 euros for the third dose). Additionally, reminder text messages were sent for this group: “(Name), don’t forget your HPV jab on (day) at (time) at the (venue). Thank you” [48].

#### 3.3.4. Reminder Intervention/Strategy

Chao et al. proposed and evaluated a reminder intervention, involving a letter sent to patients; the letter included a message reminding patients of their vaccination schedule, the date of the first vaccination, and the recommendation to continue with the vaccination. It also provided contact details, such as phone number for scheduling vaccination. Additionally, information about the vaccine was sent on this occasion. The letter was sent on behalf of the family doctor and was tailored to address the patient directly in a language appropriate for their age. For adolescents aged 9–11, the letter was sent to their parents, while adolescents and young women aged 12–26 received the message personally [49].

### 3.4. Outcomes of the Original Studies

A total of 68.57% of the included original studies (24 out of 35) evaluated the knowledge and/or awareness of participants about HPV infection, HPV vaccine and/or cervical cancer, 45.71% (16 out of 35) assessed intentions/willingness to get the vaccine, and 54.28% (19 out of 35) analyzed the actual HPV vaccine uptake (initiation and/or completion of vaccination). Some of the studies evaluated attitudes, beliefs, perceptions, and acceptability of the intervention.

Most of the interventions applied in the original studies (25 out of 35 studies—71.43%) presented results in comparison to a control group.

#### 3.4.1. Knowledge about HPV Infection, HPV Vaccine and/or Cervical Cancer

**Figure 2:**
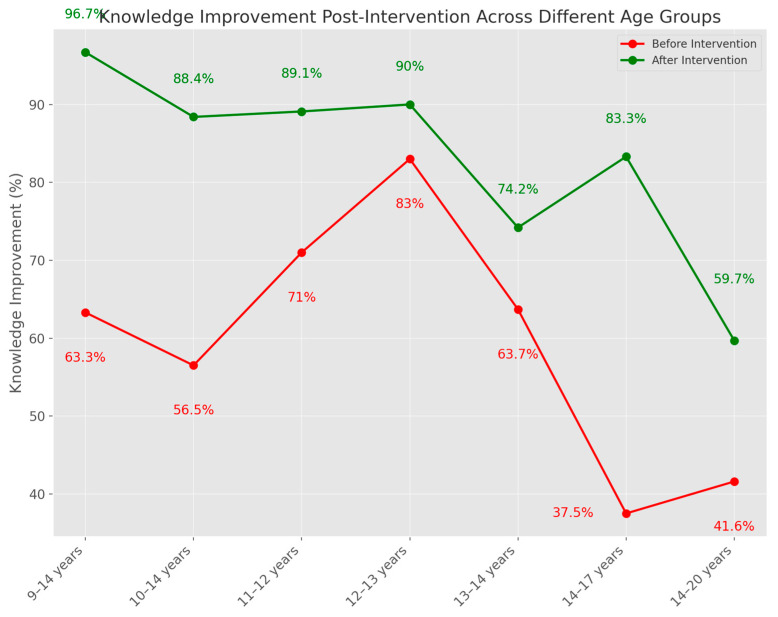
Knowledge improvement post-intervention across different age groups.

The level of knowledge was higher after all interventions, regardless of the approach. Also, significant differences were reported between intervention and control groups. Some studies (Zhang et al., Ifediora et al., Liu et al., Davies et al.), reported that knowledge remained high for all the follow-up periods [18,22,25,46] (Figure 2).

#### 3.4.2. Intentions/Willingness to Get the Vaccine

Out of the 16 studies that evaluated intentions toward vaccination, 14 reported increased intentions after intervention. In contrast, Wegwarth et al. reported a reduced intention to get vaccinated in girls who read the balanced leaflet by 8%, while intention increased by 40% in adolescents who received the unbalanced leaflet [35]. Also, Gotvall et al. reported no significant differences in intentions after the educational intervention: 7 adolescents (8%) in IG and 11 in the CG (11%) [20].

**Figure 3:**
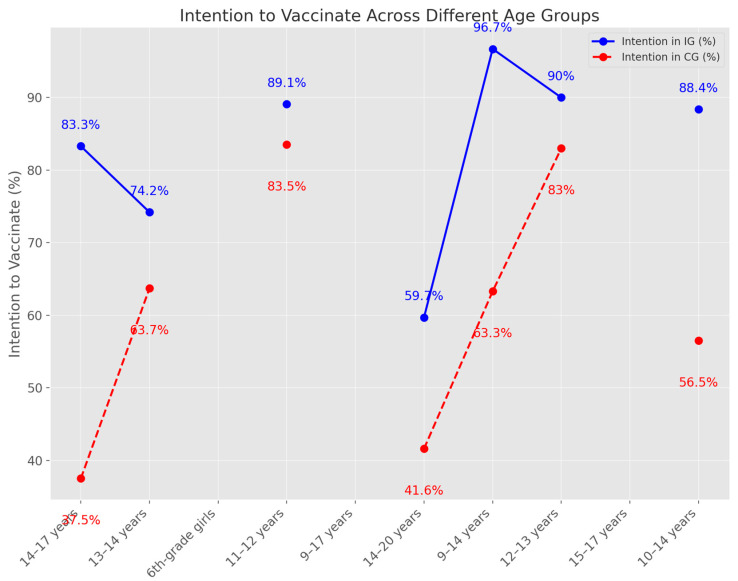
Vaccine intention across age group.

The intention to vaccinate among different age groups showed varied results. In the 14–17 years age group, there was a higher intention to vaccinate, with 83.3% compared to 37.5%. Among 13–14-year-olds, the intention was higher in the intervention group (IG) at 74.2% versus 63.7% in the control group (CG), with a *p*-value of less than 0.001. For 6th-grade girls, the intention to vaccinate decreased in the IG (using a balanced leaflet) and increased in the CG (using an unbalanced leaflet). The 11–12 years age group showed an increase in intention from 71% to 89.1%, with 83.5% overall. A significant increase in intention to vaccinate was observed in the 9–17 years age group. For those aged 14–20 years, the intention increased from 41.6% to 59.7%, and for 9–14 years, the increase was from 63.3% to 96.7%. In the 12–13 years age group, the intention to receive the vaccine was 90% compared to 83%, with a *p*-value of 0.015. Among 14–17-year-old girls, the intention to vaccinate was higher, with a ratio of 4 to 1. In the 15–17 years age group, there was a 2.368 points improvement in vaccination intention in the IG compared to the CG. For those aged 14.74 years, there was an 11.3% increase in intention to vaccinate post-intervention. Finally, for the 10–14 years age group, the willingness to vaccinate increased from 56.5% to 88.4%, with a *p*-value of less than 0.001 (Figure 3).

#### 3.4.3. Vaccine Uptake

A total of 19 studies reported vaccination rates (initiation and/or completion) after the intervention. A total of 11 of them (57.89%) outlined improvement in HPV vaccine uptake post-intervention.

Chao et al. (reminder letters approach) reported significant statistical differences in HPV vaccine series completion between the intervention and the control group (56.4% versus 46.6%, *p* < 0.001) [49].

Costantino et al. (lesson held by medical professionals and researchers and slide presentation) stated that 69.1% of the adolescents that participated in the formative interventions and who had not previously received any dose of the HPV vaccine, were vaccinated [19].

The study conducted by Grandahl et al. (face-to-face health interviews delivered by school nurses, flipcharts, leaflets) shows an increase in the vaccination coverage for the intervention group (from 52.5% to 59%, *p* = 0.02), with no modification in the vaccination rates for the control group [8]. Poscia et al. also reported a significant increase in HPV immunization rates (+11%, *p* = 0.002) after a school-based educational intervention [29].

After an educational intervention that targeted aside from adolescents, parents, and also healthcare providers, Suryadevara et al. reported increased HPV vaccine initiation and completion. The rise in the vaccine series completion rates for all practices involved in the study ranged between 12 and 20% for 11–12-year-olds and between 7 and 23% for 13–18-year-olds [38].

Peer education interventions had promising results in increasing HPV vaccine uptake. Thus, Ferrara et al. reported higher vaccination rates after a school-based peer health education intervention (from 14.1% to 17.5%) [43]. Also, Rane et al. reported a higher uptake after involving student champions in educational programs (the mean HPV uptake was 14% higher in intervention SBHCs, with a 33% higher mean completion rate) [44].

Cates et al. reported a higher vaccine initiation (22% vs. 15%, *p* = *0*.31) and completion rate (9% vs. 2%, *p* = *0*.10) after exposure of parents-adolescents dyads to a video game [26]. A total of two adolescents (2%) received the HPV vaccine 6 months after an educational intervention under the form of a comic book in the study led by Shin et al. [28].

In the study conducted by Esposito et al. (website and lecture), the authors reported that 6% of the adolescents in the CG, 4% in the website group, and 7.6% in the website + lecture group were vaccinated (*p* = 0.27) [7].

After a multicomponent (educational and reminder) intervention (Mohanty et al.), 176 doses of the HPV vaccine were administered to 152 adolescents. The reminder-recall letters sent directly to adolescents were considered by the authors more successful in convincing the adolescents to get the vaccine than the Facebook page [45].

Another multicomponent (educational, financial incentives and reminder system) intervention (Mantzari et al.) had promising results, with a significantly increased (10%) initial uptake and completion of the HPV vaccine [48].

Di Clemente et al. also reported, in a multicomponent (educational and reminder) intervention, that 12 participants in each group (intervention and control) received the first dose of the HPV vaccine, but the acceptability for the second and third dose was higher in the IG (26 doses vs. 17 doses) [47].

Gotvall et al. reported that, after an educational intervention (lesson and website), 15 girls (16%) in the intervention arm and 15 girls (14%) in the control group received the HPV vaccine, the difference not being significant statistically (*p* = 0.667) [20].

Ortiz et al. (Facebook page) also reported a not significant increase in HPV vaccination after the intervention. Lee et al. [34] (narrative video) found no difference in vaccine initiation rates between the intervention and control groups (2 vs. 2) [41].

Wegwarth et al. also did not report any difference between the two groups (balanced and unbalanced) in the actual vaccine uptake; on the other hand, for 97% of the cases in the balanced leaflet group and only 60% of the cases in the unbalanced leaflet group, the intention matched the actual decision [35].

After a complex multicomponent (educational and logistical) intervention, Davies et al. reported a non-significant difference in favor of the IG for each HPV vaccine dose: dose 1–0.8%; dose 2–0.2%; dose 3–0.5% [46].

In the study conducted by Sanderson et al., that targeted parents, adolescents, and healthcare providers, 45.4% of patients in the intervention group received the HPV vaccine during the initial visit, compared to 32.9% in the usual care arm, but the authors reported that the results were not significant after adjusting for mother’s education and child’s age. Also, the study reported a significantly lower completion of the vaccine series by 12-month follow-up in the intervention arm versus the usual care arm [33].

**Figure 4:**
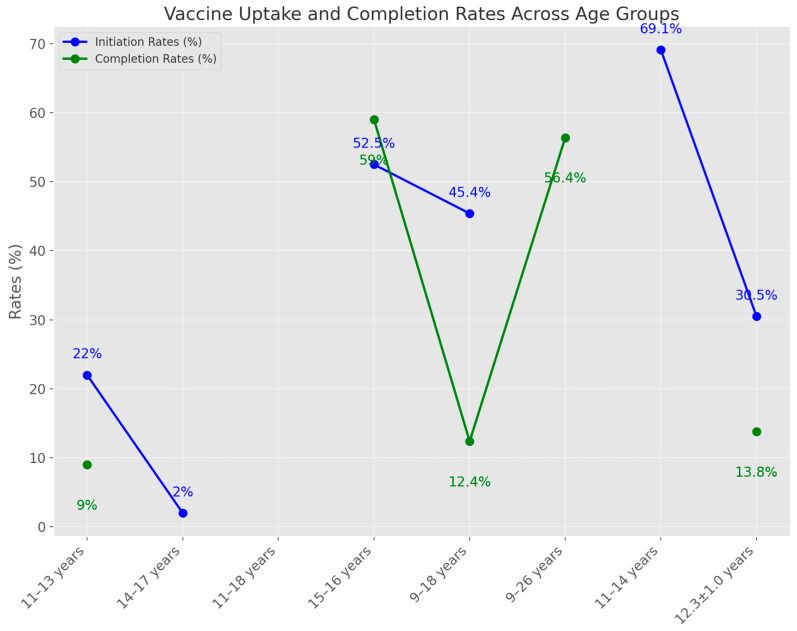
The vaccine uptake and completion rates post-intervention across different age groups.

The vaccine uptake (Figure 4) and completion rates among various age groups revealed notable differences. For the 11–13 years age group, the vaccine initiation rate was 22% compared to 15%, and the completion rate was 9% versus 2%. In the 14–17 years group, 2% of adolescents received the vaccine within six months post-intervention. Among 11–18-year-olds, 44 adolescents were vaccinated: 17 in the control group (CG), 9 in the website group, and 18 in the website plus lecture group. In the 15–16 years age group, vaccination rates increased from 52.5% to 59% post-intervention, with a *p*-value of 0.02. For those aged 9–18 years, 45.4% in the intervention group (IG) received the HPV vaccine during the initial visit compared to 32.9% in usual care, although the completion of the three-dose vaccine series was lower in the IG (12.4% vs. 18.0%) (Figure 4).

In the 9–26 years age group, the HPV vaccine series completion was 56.4% in the IG versus 46.6% in the CG, with a *p*-value of less than 0.001. Among 11–14-year-olds, 69.1% were vaccinated post-intervention. For 16-year-olds, 15 girls in both the IG and CG1 received vaccination. In the 11–18 years age group, the initiation and completion rates of the HPV vaccine series increased by more than 10% in three practices and by 5% in five practices. For those aged 16–18 years, initial uptake and completion of the HPV vaccine increased by 10% with financial incentives. Among 11–18-year-olds, the mean HPV uptake was 14% higher in intervention school-based health centers (SBHCs) compared to controls, with an initiation rate 11% higher and a completion rate 33% higher. Finally, in the 12.3 ± 1.0 years age group, a higher proportion of girls in the IG received the vaccine (30.5% vs. 13.8%, *p* = 0.003), with a significant increase in HPV immunization rates by 11%, and a *p*-value of 0.002 (Figure 4).

### 3.5. Outcomes of the Systematic Reviews

Acampora et al. observed that combined interventions/multimodal strategies, such as education, information, and communication, along with other types of interventions leads to more effective and sustainable results; the greater the number of interventions, the better the outcomes. Promising results have been obtained with peer-to-peer educational interventions, focus groups with adolescents and/or parents, and doctors; watching narrative videos about HPV vaccination influenced vaccination intention in the intervention groups. Reminder strategies directed to adolescents/parents were well received by adolescents, who, according to the analyzed data, prefer text message or email interventions and resulted in a significant increase in vaccination initiation in three studies out of five and an increase in complete vaccination rates in two studies; however, one study did not show an increase in complete vaccination rates after the intervention [11].

Another systematic review (Abdullahi et al.) highlighted that educational interventions improved HPV vaccination rates in adolescents compared to standard practice; thus, when adolescents and their parents received information and education about vaccination, more adolescents were vaccinated. The review does not show an increase in vaccine acceptability or it was insignificant after offering financial incentives [14].

According to Walling et al., informational and educational interventions that targeted both individuals and communities resulted in increased acceptance of vaccination and higher vaccination rates during the intervention. However, the effects of this type of intervention decreased over time, suggesting the need for sustained campaigns in conjunction with other types of interventions. Reminder interventions have been found to be highly effective. Interventions aimed at healthcare staff had a significant impact on vaccination initiation, while those focused on patients were more successful in ensuring completion of the vaccination schedule. Therefore, the authors noted that healthcare professionals can be a major barrier to vaccination initiation, often due to lack of recommendation or unsupported recommendation, whereas patient and family decisions more often affect completion of the vaccination. The most effective results were achieved through multidirectional interventions that targeted both medical professionals and patients. The authors also emphasized that social interventions, such as increasing accessibility by reducing costs or organizing new vaccination sites, can significantly contribute to higher vaccination rates [12].

In another study, the researchers pointed out that educational interventions have a greater impact on the intention to vaccinate in adolescents and young adults compared to parents. All studies involving these age groups demonstrated a significant improvement in attitudes and perceptions towards vaccination immediately after the intervention, regardless of the format and content of the educational materials. However, there is no evidence to suggest that these attitudes will be sustained in the long term and lead to increased vaccination rates [15].

The analysis of three studies that included only patients in another review yielded conflicting results. Two studies reported significantly increased vaccination rates after the intervention, while the third showed no differences in vaccination rates between the two groups. On the contrary, a decrease in vaccination rates was reported, with almost a two percent difference between the intervention and control groups [16].

After the intervention, all 15 studies reviewed by Flood et al. showed a significant improvement in knowledge, attitudes, and perceptions regarding vaccination as a cancer prevention method, regardless of the intervention’s content, presentation, or duration. The analysis suggests that interventions targeting behavior change are the most effective. Peer education showed good results, as adolescents feel more comfortable discussing with their peers. This study also indicates that while knowledge levels may decrease approximately 6 months after the intervention, they still remain high compared to pre-intervention levels. However, continuous interventions and follow-ups over a longer period are necessary to assess behavior changes [13].

## 4. Discussion

### 4.1. Barriers to HPV Vaccination

Knowledge about vaccine-preventable diseases, the risks associated with them and the availability of correct information about vaccines and vaccination, can positively influence the acceptability of immunization not only by parents but also when we talk about adolescents. One of the main reasons why teens choose not to get vaccinated is due to a lack of information. Adolescents consider family, school, and medical staff as the main and most credible sources of information on vaccination [50].

However, while vaccine hesitancy towards vaccination is often attributed to a lack of knowledge, there are multiple reasons that can lead to doubts and concerns about vaccines and vaccination among parents, adolescents, and even medical professionals [51,52,53,54].

Concerns about the safety of the vaccines and possible side effects are cited in most studies as a decisive factor in the development of hesitant behavior. Not only parents, but also teenagers are worried about potential adverse reactions following vaccination [55].

The belief that the child is not at risk of infection leads some parents to believe that HPV vaccination is not necessary [56]; the same is true for adolescents [50,57]. Beliefs that HPV vaccination could change adolescent sexual behavior can also hinder vaccine acceptability [57].

The influences of the social environment are important; the relationship with the doctor, family, and close ones, affects the behavior of both parents and adolescents; thus, the negative attitude related to vaccination and the parents’ recommendation not to get vaccinated decisively influences the adolescents’ intention [58].

The lack of trust in the authorities and medical personnel, and the conspiracy theories accentuate the hesitation of parents. Distrust of vaccines in general, concerns about vaccine efficacy [57], doubts about the benefits of vaccines, and low perception of disease risk and disease severity are important barriers to vaccination [51,52].

The doctor’s failure to recommend vaccination significantly impacts its acceptability among parents and adolescents [57,58]. Also, the fear of injection and the pain associated with the administration of vaccines are potentially discouraging factors in the acceptability of immunization by adolescents [50].

Media, social media, the internet, health policies that regulate vaccination influence vaccination-related behavior [51,54].

Lack of access to vaccination: the cost of the vaccine, the time required, the distance, are important barriers to vaccination [51,54]. In a study that evaluated the obstacles to implementing HPV vaccination programs in low- and middle-income countries, the most significant barrier was the lack of funds, resources, and political support at the government level. At the individual level, the perceived cost/benefit ratio was reported as the most important barrier [59].

Sociodemographic factors such as age, gender, level of education, income, but also personal beliefs about health and prevention, emotions, culture, and traditions can influence vaccination decisions. Relying on alternative methods instead of vaccination is increasingly recognized as a cause of vaccine-hesitant behavior [53].

Interventions targeting adolescents for HPV vaccination vary significantly across countries due to factors beyond age, such as healthcare infrastructure, government policies, cultural attitudes, socioeconomic conditions, and the role of educational systems. In high-income countries with developed healthcare systems, such as the U.S., Canada, and many European nations, interventions often focus on school-based vaccination programs, where vaccines are administered within educational institutions, coupled with reminders through electronic health records or mobile apps. In contrast, low- and middle-income countries (LMICs) often rely on community-based initiatives, such as mobile health clinics and public health campaigns, to reach adolescents, particularly in rural or underserved areas [60].

Government policies also play a crucial role in shaping interventions. In countries like Australia, where HPV vaccination is mandated or highly recommended, large-scale public health campaigns and school-based programs have high coverage and success rates. However, in countries where vaccination is voluntary, interventions often need to focus on raising awareness and combating vaccine hesitancy. Cultural and social attitudes further influence the effectiveness of these interventions. In conservative societies, such as in parts of the Middle East and Southeast Asia, addressing concerns around the HPV vaccine’s association with sexual activity requires careful messaging, often reframing it as a tool for cancer prevention to avoid cultural resistance [61].

Socioeconomic factors also impact vaccination strategies. In wealthier nations, logistical barriers, such as access to healthcare facilities, are often the primary challenge, and interventions focus on improving convenience and adherence. In lower-income countries, the cost of the vaccine is a significant barrier, with interventions often relying on subsidies or international aid to make vaccines accessible. Additionally, digital and social media platforms play an increasing role in high-tech nations like South Korea and the U.S., where social media campaigns are used to reach adolescents directly, while in countries with limited digital access, traditional media and community outreach remain the dominant strategies [62].

Educational systems are another critical factor. Countries with robust school-based health programs, such as the UK and Australia, can effectively deliver vaccines within schools, reducing the need for clinic visits. However, in countries with weaker educational infrastructures or lower school attendance rates, interventions often depend on community health workers and mobile vaccination units to reach adolescents. Furthermore, parental involvement varies across regions; in some countries, parents have significant control over vaccination decisions, requiring interventions to focus on educating both parents and adolescents, while in others, adolescent autonomy is prioritized, making it essential to target information directly to young people [63].

Overcoming these barriers is a difficult task, due to the multiple factors that can influence an individual in the process of making a decision about vaccination.

In this review, studies that present interventions aimed directly at adolescents were examined. The most commonly described interventions were educational, but multicomponent and reminder-based interventions were also identified.

### 4.2. Impact of Interventions on HPV Knowledge

Wegwarth et al. emphasize the importance of providing accurate, transparent, and balanced information in order to improve knowledge about HPV vaccination, including both the benefits and the potential risks. Unbalanced medical information can lead to misinformation, while accurate information increases understanding of the topic [35].

Educational interventions improved adolescents’ knowledge scores in the selected studies. In some cases, knowledge levels remained high even one year after the intervention, when follow-up was conducted [18,22,25]. Not all the studies reported if the differences in knowledge scores between the IG and the CG were statistically significant. Educated adolescents exhibited a better understanding/awareness of HPV infection, vaccination, and cervical cancer. They also demonstrated a better perception of the benefits of vaccination, its role in protecting against cancer [27,40] and the risks associated with HPV infection and related diseases [8], and a higher awareness of symptoms of early cervical cancer [21]. Adolescents with adequate knowledge and attitudes were more likely to adhere to vaccination [39].

Peer education proved to be highly effective in rapidly and significantly improving knowledge about HPV [2,43].

Multicomponent interventions generated awareness [45] and better knowledge when compared to the control group that persisted months after intervention [46].

### 4.3. Impact of Interventions on HPV Vaccination Intention

Out of the studies that reported intention/willingness to get vaccinated after the intervention, no matter the type, only one reported lower intention, in the group that received balanced information, a group that probably perceived the risk of HPV-associated cancer as being low; on the other hand, for 97% of the cases in the balanced leaflet group, the intention matched the actual decision, leading to the conclusion that the balanced, honest information increases informed decisions about whether to be vaccinated and did not affect actual uptake [35].

Intention does not always match decision/uptake. Behaviors are more difficult to change than knowledge. This is also true in the case of adolescents. In order to modify behavior, interventions must be specific, and tailored to the individual needs, and they must be constantly, and repeatedly implemented. The information we transmit is important, but also the way we propagate it is important.

### 4.4. Impact of Interventions on HPV Vaccination Uptake

Educational interventions followed by vaccine uptake evaluation, showed variable data. A total of two studies reported a statistically significant increase in HPV immunization rates after interventions [8,29]. A total of nine studies also reported an increase in HPV vaccine uptake, but the differences between the intervention and the control group were not statistically important or were not mentioned as being statistically significant [7,19,20,26,28,38,41,43,44]. The other two studies reported no difference between groups in vaccination rates [34,35]. Sanderson et al. reported higher vaccine uptake at the initial visit but significantly lower completion of the three-dose vaccine series in the intervention arm [33].

Only one study out of those that proposed a multicomponent intervention reported no significant difference in favor of the intervention group for any of the HPV vaccine doses [46]. Another one [47] showed higher compliance for the second and third dose and the remaining two studies that used education + reminder and education + reminder + financial incentives also had a very good impact on vaccination rates, initiation and completion being significantly increased after the intervention [45,48]. The single study that used the reminder strategy also reported higher HPV vaccine series completion after intervention and the effect of the intervention appeared to be stronger in girls aged 9–17 years compared with young women aged 18–26 [49].

### 4.5. Adolescent HPV Education

These results emphasize the need for standardized and extended school-based interventions, for expanding the duration of the intervention, improving the messages, for a longer follow-up, for better development of multicomponent strategies, and also for wider implementation of reminder systems in order to enhance vaccination.

Adolescent education is important, can influence vaccination rates, and should be performed in a well-structured and grounded framework, as parents are also not well-informed enough to be an important source of information or may not feel comfortable discussing HPV infection and sexually transmitted infections [6]. Also, educational interventions targeting adolescents must be adapted to their age and its challenges [64] and should address the main barriers to vaccination, including low knowledge and awareness, misinformation about HPV vaccination, and low perception of the risk of infection [65].

The school is generally considered an ideal location for both educating adolescents and administering vaccinations. Constant educational interventions conducted in schools, especially interactive ones, whether through face-to-face interactions, or dissemination of information via brochures, printed materials, or PowerPoint presentations, can significantly influence attitudes towards vaccination [7].

The implementation of health education programs in schools and the promotion of preventive behavior can contribute, in the long term, to reducing the incidence rates of cervical cancer. Ultimately, this is the goal of these interventions [32].

Marek et al. reiterate the idea that there is a need for continuous education, adapted to specific age groups. The effectiveness of the interventions depends on their duration and requires periodic booster sessions [21].

Liu et al. also recommend integrating health education into existing sex education programs and offering it constantly at short intervals to maintain the information and enhance the effect [25].

Pre-vaccination educational interventions in schools can enhance vaccination knowledge and awareness, boost confidence in vaccines, and improve confidence in making positive vaccination decisions [4,5,6].

Teachers can play a crucial role in training and educating adolescents, as well as in conveying information to parents, particularly in the context of school-based vaccination programs [14].

Thus, it is becoming increasingly clear that it is crucial to include education on health, HPV infection, vaccines, and vaccination in school curricula, especially in countries with low socioeconomic levels and high incidences of cervical cancer. It is important that this education is continuous and complemented by assessments of knowledge levels [18].

The inclusion of health education classes in school curricula, as well as courses on immunization, could improve the level of knowledge and implicitly, the acceptability of vaccination [64,65,66].

Other studies also stress the significance of including HPV education in the secondary school curriculum in countries where this has not been performed yet. They also recommend introducing a course or chapter on sexually transmitted diseases in high school biology classes to raise awareness among adolescents [32].

Peer education is an effective intervention for addressing issues related to sexually transmitted infections and more. It involves well-trained and motivated young people leading educational activities with their peers to increase knowledge, change attitudes, and develop skills for practicing healthy behaviors. Although it is relatively easy to implement, this strategy requires careful planning, coordination, supervision, and resource allocation [43].

Utilizing social media platforms for public health interventions can be highly effective in increasing awareness. This type of communication is easy, cost-effective, and reaches a wide audience. It allows for real-time feedback and encourages message sharing among users, leading to greater dissemination and impact [67].

Social media platforms have the potential to generate interest in HPV infection and vaccination among adolescents, and they can have a positive influence on the decision-making process concerning vaccination [45].

Conversely, technology-based educational programs can also have a significant impact on adolescents [7].

Digital technology, specifically mobile health (mHealth), involves the transmission of health information through mobile devices. It can be used to increase vaccine acceptability by providing easy access to information, support, and guidance, and facilitating rapid communication [68].

### 4.6. Study Limitations and Strengths

The first limitation is that while the Mixed-Methods Appraisal Tool (MMAT) was used, it may not fully capture the nuances of quality for all study designs, potentially affecting the consistency of assessments. The inclusion of diverse study types, such as qualitative, quantitative, and mixed-methods studies, introduces heterogeneity, complicating the synthesis of results and limiting unified conclusions. Publication bias may also have influenced the findings, as unpublished studies or grey literature were not systematically included. Additionally, the geographical and cultural concentration of studies may restrict the generalizability of the results to broader populations. Many studies lacked long-term follow-up, limiting the assessment of sustained intervention effects. Furthermore, variability in the reporting of interventions across studies makes direct comparisons difficult. Finally, some studies had small sample sizes, which could reduce statistical power and generalizability. The strengths of this study lie in its identification of effective strategies and interventions to improve HPV vaccination rates among adolescents, offering valuable insights for future public health initiatives, although it is limited by the exclusion of non-English research. Addressing these limitations in future research will enhance the robustness and applicability of findings.

## 5. Conclusions

Even though implementing educational interventions focused on adolescents requires resources, time, and costs, even short-term interventions with minimal resources can have a significant impact. This impact can be measured in terms of increased knowledge and improved attitudes about HPV and vaccination, which can result in higher vaccination rates [69,70]. For the development of these strategies, it is important to consult all parties involved in the decision. Understanding behaviors and integrating scientific theories to change them is crucial for a multimodal and multidirectional approach [71,72].

## Figures and Tables

**Table 1:** The quality assessment results for studies included in a review.

Study Type	Criteria Met (out of 5)	Quality Judgment	Number of Studies
Qualitative	3/4	Moderate quality	5 (12.20%)
Qualitative	4/5	High quality	8 (19.51%)
Quantitative RCT	3/5	Moderate quality	6 (14.63%)
Quantitative RCT	4/5	High quality	3 (7.32%)
Mixed Methods	3/5	Moderate quality	6 (14.63%)
Mixed Methods	4/5	High quality	13 (31.71%)
Total			41 (100%)

RCT = randomized controlled trials; Met = met the criteria.

**Table 2:** Methodology for the selection of the studies.

Methodology for the Selection of the Studies		
Keywords, separated by OR operator	“HPV vaccination”, adolescents/teenagers/teens”, ”interventions/strategies”,	Total records
Databases searched	PubMed, Web of Science, Scopus	
Records identified	PubMed	3745
	Web of Science	536
	Scopus	885
Records after duplicate removal	PubMed + Web of Science + Scopus	3353
Records excluded	PubMed + Web of Science + Scopus	3124
Full test records evaluated for inclusion	PubMed + Web of Science + Scopus	229
Full text excluded	PubMed + Web of Science + Scopus	188
Records included in the final review	Original articles (35) + Systematic reviews (6)	41

HPV = human papillomavirus.

## Data Availability

All the data processed in this article are part of the research for a doctoral thesis, being archived in the aesthetic medical office, where the interventions were performed.
